# Influence of UiO-66(Zr) Preparation Strategies in Its Catalytic Efficiency for Desulfurization Process

**DOI:** 10.3390/ma12183009

**Published:** 2019-09-17

**Authors:** Alexandre M. Viana, Susana O. Ribeiro, Baltazar de Castro, Salete S. Balula, Luís Cunha-Silva

**Affiliations:** REQUIMTE/LAQV & Department of Chemistry and Biochemistry, Faculty of Sciences, University of Porto, 4169-007 Porto, Portugal

**Keywords:** porous metal-organic frameworks, UiO-66(Zr), solvothermal synthesis, microwave assisted synthesis, heterogeneous catalysis, oxidative desulfurization

## Abstract

Porous metal-organic framework (MOF) materials UiO-66(Zr) obtained by solvothermal and microwave advanced synthesis (MWAS) procedures were characterized, and their catalytic efficiency was investigated for oxidative desulfurization (ODS) processes using a multicomponent model diesel containing benzothiophene and dibenzothiophene derivatives. The preparation parameters as the cooling time after oven use in the solvothermal procedure, and also the reaction time in the MWAS method seemed to play an important role in the catalytic performance of the UiO-66(Zr) material, as well as in its recycle capacity. The material prepared by the solvothermal procedure with a fast cooling time showed the best catalytic performance (desulfurization efficiency of 99.5% after 3 h). However, the application of the UiO-66(Zr) material prepared by the MWAS method (desulfurization efficiency of 96% after 3 h) conciliated a higher number of advantages, such as shorter reaction time preparation (15 min) and high catalytic activity for a higher number of reaction cycles. The UiO-66(Zr) prepared by the MWAS method was used for the first time in an oxidative desulfurization process, and according to the catalytic results obtained (high recycle capacity and stability) and shorter reaction time preparation, seems to be a promising material for industrial application.

## 1. Introduction

The incessant need for energetic and environmental sustainable development has promoted high attention to fossil fuel consumption and production, since it is still the main source of energy for many purposes, namely transportation. The combustion of these fuels is associated with emissions of different sulfur oxides and fine particles of metal sulfates to the atmosphere, originated from sulfur content present in crude oil [1]. These emissions are responsible for many environmental problems related to acid rain and associated with public health issues, which have stimulated reasonable international legislative regulation over the petrochemical industry [2]. Furthermore, those sulfur-containing compounds are also unwanted in industrial refining processes, since they induce corrosion of equipment and catalyst deactivation. Nowadays, hydrodesulfurization (HDS) is the main process used for sulfur removal from fuels in the industrial context [3]. HDS is an effective process for the elimination of different types of sulfur content, such as mercaptans, sulfides, disulfides and their derivatives. Unfortunately, this method is always associated with catalytic processes at high temperatures and pressure conditions, which require great consumption of energy and expensive hydrogen gas. Furthermore, HDS is not an effective method for the removal of heterocyclic sulfur compounds such as thiophenes and its derivatives, which are the other main groups of sulfur content present in fuels [4].

The oxidative desulfurization process is a promising method for the desulfurization of diesel fuels that has been the object of extensive research efforts in recent years as the answer for the need of a more efficient and sustainable method for desulfurization [5]. Oxidative desulfurization allows the removal of even the most refractory sulfur content present in fuels, namely thiophenes and dibenzothiophene derivatives, under low conditions of temperature and pressure and without requiring hydrogen consumption. An oxidative desulfurization process is carried in two steps: (i) the oxidation of sulfur-containing compounds, during which an oxidizing agent, such as hydrogen peroxide, donates one or two oxygen atoms to convert those compounds to their corresponding sulfoxides or sulfones; (ii) and extraction, which can be done with an adequate extraction solvent [6].

The efficient oxidative desulfurization process is made possible while resorting on appropriate catalysts to aid the oxidation step, and many different heterogeneous catalysts have been considered for this purpose [6]. Curiously, only a limited number of metal-organic framework (MOF) materials have been proved to be efficient and highly selective catalysts for oxidative desulfurization processes [7,8,9]. The MOFs are coordination polymers assembled by two different building blocks: Metal centers and organic ligands. These hybrid materials, mostly crystalline and porous, can be uni-, bi- or tridimensional and are very popular for their diverse structural arrangements and easily tunable properties. The interest associated with these materials also comes from their wide range of potential applications in gas storage and separation [10], as adsorbents [11], sensors [12] and catalysts [13].

**Figure 1:**
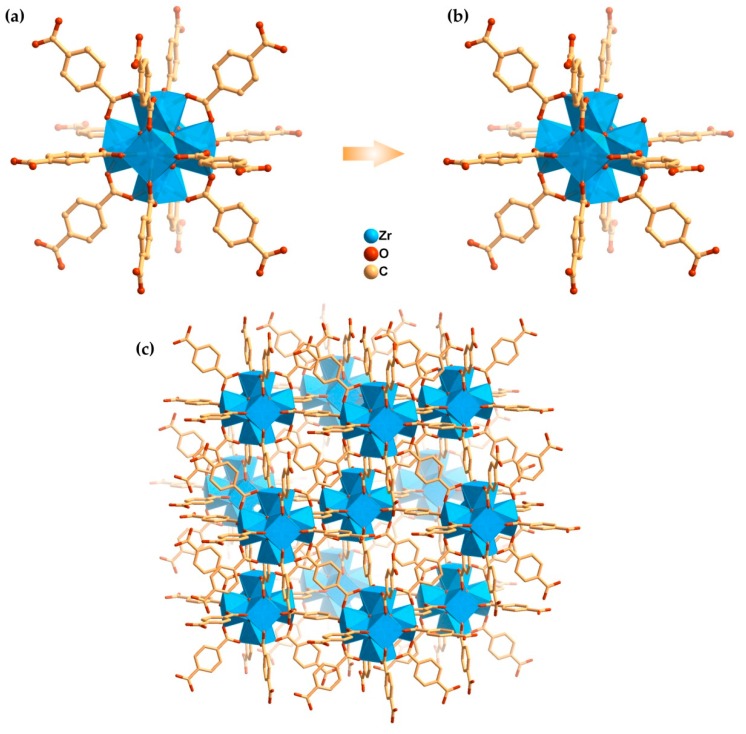
(**a**) Defect-free [Zr_6_O_4_(OH)_4_] cluster centre coordinated by 12 benzenedicarboxylate (bdc) ligands in the structure of Uio-66(Zr) and (**b**) the centre with a defect site; (**c**) 3D porous structure of MOF UiO-66(Zr). H-atoms were omitted for clarity proposes. Images prepared from the CIF file obtained from CDS with code RUBTAK04 and reported by Trickett and co-workers [7].

The UiO-66(Zr) is a porous MOF based on [Zr_6_O_4_(OH)_4_(CO_2_)_12_] secondary building units, prominent for high surface area and exceptional chemical, mechanical and thermal stability (Figure 1). Furthermore, it is well-known by the ability to support structural defects around the metal cluster centre without losing the framework integrity, enhancing its properties and potential applicability as a sensor [14,15], adsorbent [16,17] and catalyst [18,19]. Following our interest in the development of MOF-based materials as heterogeneous catalysts for oxidation reactions, [20,21,22,23,24] in particular oxidative desulfurization processes of model and real diesel, [10,25,26,27,28,29] the distinct samples of the MOF UiO-66(Zr) prepared by solvothermal and microwave assisted synthesis (MWAS), were applied as catalysts in the oxidative desulfurization processes of model diesel. The influence of the preparation method and procedure parameters in the oxidative desulfurization performance was evaluated.

## 2. Experimental Section

### 2.1. Materials

All the reagents used in the preparation of the MOF materials [zirconium(IV) chloride (Aldrich, St. Louis, MO, USA), titanium(IV) chloride (Aldrich), benzene-1,4-dicarboxylic acid (Aldrich), *N*,*N*-dimethylformamide (DMF, Aldrich), and ethanol (Fisher, Hampton, NH, United States) were used as received without further purification, and those employed in the desulfurization studies [1-benzothiophene (1-BT, Merck, Kenilworth, NJ, USA), dibenzothiophene (DBT, Aldrich), 4-methyldibenzothiophene (4-MDBT, Aldrich), and 4,6-dimethyldibenzothiophene (4,6-DMDBT, Alfa Aesar GmbH & Co KG, Haverhill, MA, USA), acetonitrile (MeCN, Panreac, Barcelona, Spain), ethyl acetate (Fisher), hydrogen peroxide (H_2_O_2_ 30%, Aldrich), *n*-octane (Acros Organic), xylene (Aldrich), and tetradecane (Aldrich) were also used as received. The untreated diesel sample was supplied by Galp containing approximately 2300 ppm of sulfur without any previous treatment.

### 2.2. Characterization Methods

The Fourier transform infrared spectra were acquired on the attenuated total reflectance operation mode (FTIR-ATR) on a Perkin Elmer FT-IR System Spectrum BX spectrometer (typically: range 4000–400 cm^−1^, 2 cm^−1^ resolution and 32 scans), and all the representations are shown in arbitrary unities of transmittance.

The powder X-ray diffraction (PXRD) patterns were obtained at room temperature on a Rigaku’s Smartlab diffractometer working with a Cu radiation source (λ = 1.540593 Å) and in a Bragg-Brentano θ/2θ configuration (45 kV, 200 mA). The intensity data were collected by a step-counting method (step 0.01°), in continuous mode, in the ca. 3 ≤ 2θ ≤ 60° or 3 ≤ 2θ ≤ 90° range, and all the representations are shown in arbitrary unities of intensity.

The scanning electron microscopy (SEM) images and electron dispersive X-ray spectroscopy (EDS) analysis were performed at the *Centro de Materiais da Universidade do Porto* (CEMUP, Porto, Portugal) on a FEI Quanta 400 FEG ESEM high resolution scanning electron microscope equipped with an EDAX Genesis X4M spectrometer working at 15 kV. The samples were coated with an Au/Pd thin film by sputtering using a SPI module sputter coater equipment.

The catalytic reactions were periodically monitored by GC-FID analysis carried out in a Bruker 430-GC-FID chromatograph. The hydrogen was used as a carrier gas (55 cm · s^−1^) and fused silica Supelco capillary columns SPB-5 (30 m × 0.25 mm i. d.; 25 μm film thickness) were used.

### 2.3. Synthesis of UiO-66(Zr) Samples

#### 2.3.1. Solvothermal Syntheses, UiO-66(Zr)-S

UiO-66(Zr)-S1 was prepared using an experimental procedure adapted from that previously reported [10]. An initial equimolar mixture of zirconium(IV) chloride (0.53 mmol) and 1,4-benzenedicarboxylic acid (0.53 mmol) in DMF (15 mL) was prepared in an autoclave, at room temperature. The mixture was magnetically stirred for 10 min and then placed in the oven at 120 °C, for 24 h, and immediately removed from the oven to cool (fast cooling) to room temperature. The white solid was isolated by centrifugation, washed two times with DMF and ethanol, and dried overnight. The UiO-66(Zr)-S2 material was prepared and handled following the previously described steps, but the reaction mixture was left to cool in the oven for two days after the reaction was completed (slow cooling).

#### 2.3.2. Microwave Assisted Synthesis, UiO-66(Zr)-MW

The UiO-66(Zr)-MW samples were prepared by an experimental procedure adapted from that described by Vivani and co-workers. The initial equimolar solutions of zirconium(IV) chloride (0.53 mmol) and 1,4-benzenedicarboxylic acid (0.53 mmol) in DMF (15 mL) were prepared in 30 mL glass vessels, at room temperature. The mixtures were magnetically stirred for 10 min, after which the stir bar was recovered and the vessel properly caped. The mixtures were taken to the microwave synthesizer (CEM discovery SP) set at 60 W, 120 °C and 100 psi, for 60 min [UiO-66(Zr)-MW1] and 15 min [UiO-66(Zr)-MW2]. The UiO-66(Zr)-MWmod material was prepared by a similar procedure, however AcOH (2.1 mL) and deionized water (0.13 mL) were also added to the mixture, which reacted in the microwave synthesizer for 60 min with magnetic stirring. After cooling to room temperature, the three distinct solids were isolated by centrifugation, washed two times with DMF and ethanol, and dried overnight.

### 2.4. Oxidative Desulfurization Studies

The oxidative desulfurization studies were performed using a model diesel containing the most representative refractory sulfur-compounds in diesel, namely 1-benzothiophene (1-BT), dibenzothiophene (DBT), 4-methyldibenzothiophene (4-MDBT) and 4,6-dimethyldibenzothiophene (4,6-DMDBT), in *n*-octane (with a concentration of 500 ppm of sulfur from each compound). The reactions were performed under air in a closed borosilicate reaction vessel with a magnetic stirrer and immersed in a thermostatically controlled liquid paraffin bath at 50 °C. The oxidative desulfurization reactions were performed in a biphasic system composed by the model diesel and acetonitrile (MeCN) as the extraction solvent. In a typical experiment, the catalyst (15 mg, 9 µmol of Zr_6_O_4_(OH)_4_(CO_2_)_12_) was added to MeCN (0.75 mL), followed by the model diesel (0.75 mL) and the resulting mixture was stirred for 10 min. The catalytic step was then initiated with the addition of aqueous hydrogen peroxide 30% (75 μL, H_2_O_2_/S ratio of 13) to the reaction mixture. Tetradecane was used as a standard in the periodical quantification of the sulfur content by GC analysis. The UiO-66 samples were tested as heterogeneous catalysts in the extractive and oxidative desulfurization (ECODS) process. After each reaction, the solid catalyst was recovered by centrifugation, washed thoroughly with MeCN and ethanol, dried in a desiccator over silica gel and recycled. The consecutive ECODS catalytic reaction cycles were performed maintaining the reaction conditions, and preceding each one of the reactions all the materials were carefully activated by the same procedure (activation at 60 °C under vacuum for 12 h).

## 3. Results and Discussion

### 3.1. MOF Materials Characterization

**Figure 2:**
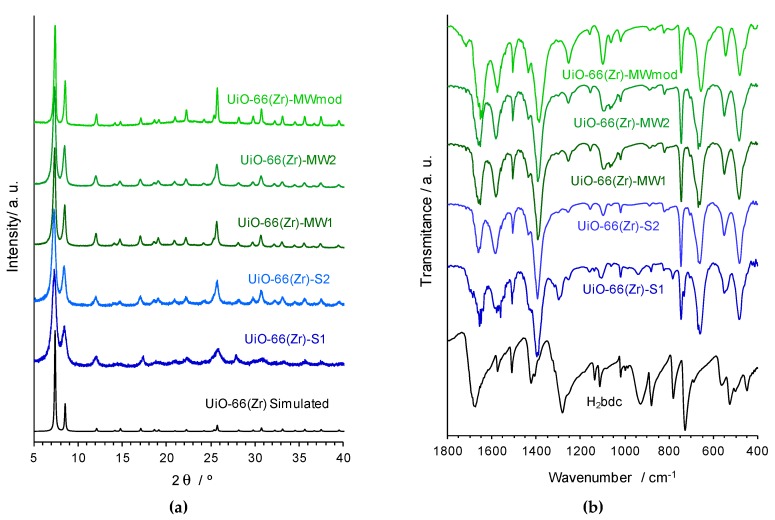
(**a**) powder X-ray diffraction (PXRD) patterns and (**b**) attenuated total reflectance operation mode (FTIR-ATR) spectra of the UiO-66(Zr) materials isolated by different synthetic methods, shown in the 2θ range 5–40° and in the wavenumber region 1800–400 cm^−1^, respectively.

**Figure 3:**
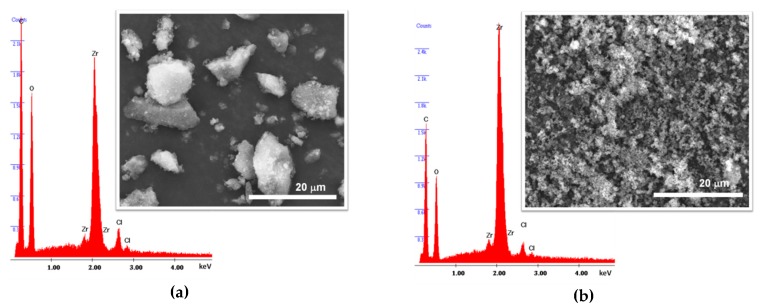
Electron dispersive X-ray spectroscopy (EDS) spectra and SEM images for the (**a**) UiO-66(Zr)-S1 and (**b**) UiO-66(Zr)-MW2 materials.

Five distinct samples of the porous MOF material UiO-66(Zr) were prepared by distinct synthetic methods, namely solvothermal synthesis [UiO-66(Zr)-S1 and UiO-66(Zr)-S2] and MWAS [UiO-66(Zr)-MW1, UiO-66(Zr)-MW2 and UiO-66(Zr)-MWmod] (see details about the synthetic procedures in the experimental section). All the isolated materials were characterized by PXRD (Figure 2a), FTIR-ATR (Figure 2b), SEM and EDS (Figure 3), allowing the confirmation of the preparation of crystalline pure phases of the porous MOF UiO-66(Zr).

The PXRD patterns are comparable with that obtained by simulation of the single-crystal X-ray diffraction data of the UiO-66(Zr) structure, both in terms of the position and relative intensities of the bands, corroborating the synthesis of this MOF by the distinct methods / conditions employed. In fact, the main diffraction peaks characteristics of the UiO-66 crystalline structure, namely at 2θ ≈ 7.4° (h, k, l = 1, 1, 1), 8.5° (2, 0, 0), 14.9 (2, 2, 2), 17.2° (4, 0, 0), 25.8° (4, 4, 2) and 31.1 (4, 4, 0) are evident in all the difractograms, even those probably less crystalline.

The FTIR-ATR spectra of the five UiO-66(Zr) samples exhibit similar characteristic peaks of this porous MOF (Figure 2b), and support their structural features namely the coordination modes of the benzecarboxylate ligand (bdc^2−^). The two intense bands in the wavenumber values at approximately 1600 and 1400 cm^−1^ are assigned to the asymmetric and symmetric stretching modes of the carboxylate group, respectively [ν_asym_(–CO_2_^−^) and ν_asym_(–CO_2_^−^)], in agreement with those values expected for the *syn,syn*-chelating coordination mode. The small band around 1510 cm^−1^ can be attributed to stretching modes of the C=C bonds in the aromatic ring, while at the lower wavenumber, the band around 740 cm^−1^ can be associated with the C–H bending mode. Furthermore, the considerable strong band around 545 cm^−1^ is consequence of the Zr–(OC) asymmetric stretching mode, and the bands around 660 and 475 cm^−1^ are most probably due to the stretching in the μ_3_-O and μ_3_-OH groups, respectively [30].

The SEM images support the evidences of the PXRD data about the preparation of crystalline materials, since all the samples prepared by solvothermal synthesis and MWAS reveal microcrystalline particles (Figure 3; for practical reasons, only SEM images for UiO-66(Zr)-S1 and UiO-66(Zr)-MW2 are shown). As expected, the materials obtained by the solvothermal method have particles considerable bigger than those of the MWAS materials. On the other hand, the EDS analysis confirms the occurrence of the predictable elements Zr and O, but also Cl in all the materials (Figure 3). The presence of Cl in the UiO-66(Zr) samples, even after the materials being meticulously washed and dried, is an apparent indication of structural defect sites in the MOF framework (Cl/Zr ratio is 0.20 for UiO-66(Zr)-S1., 0.04 for UiO-66(Zr)-S2, 0.10 for UiO-66(Zr)-MW1 and 0.07 for UiO-66(Zr)-MW2). The random absence of organic ligands (bdc^2−^) throughout the framework of materials results in charge and coordination deficiencies (open zirconium sites), which can be compensated by Cl anions [10,31]. Both the materials. UiO-66(Zr)-S1 and UiO-66(Zr)-MW2. seem to have structural defects.

### 3.2. Extractive and Oxidative Desulfurization

**Figure 4:**
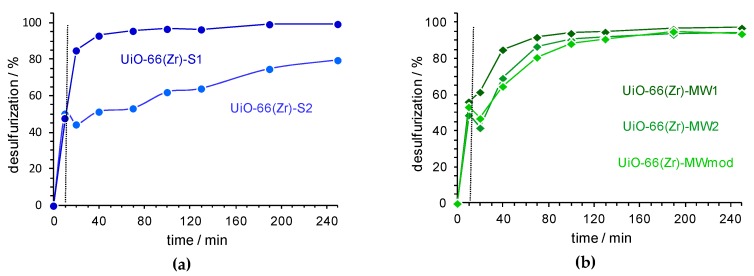
The desulfurization profiles using a biphasic model diesel/MeCN (1:1) system and H_2_O_2_ as an oxidant (H_2_O_2_/S = 13), at 50 °C: using UiO-66(Zr) catalysts prepared by the solvothermal method UiO-66(Zr)-S1 and S2 (**a**) and by MWAS UiO-66(Zr)-MW1, MW2 and MWmod (**b**). The vertical dashed lines indicate the beginning of the catalytic step in the extractive and oxidative desulfurization (ECODS) process by adding the oxidant.

The catalytic performance of the UiO-66(Zr) materials prepared by distinct methods and conditions [UiO-66(Zr)-S1, UiO-66(Zr)-S2, UiO-66(Zr)-MW1, UiO-66(Zr)-MW2 and UiO-66(Zr)-MWmod] was analyzed for oxidative desulfurization of a high-sulfur model diesel containing refractory sulfur compounds usually found in real diesel, namely 1-benzothiophene (1-BT), dibenzothiophene (DBT), 4-methyldibenzothiophene (4-MDBT) and 4,6-dimethyldibenzothiophene (4,6-DMDBT), with approximately 500 ppm of each sulfur compound (in *n*-octane). The desulfurization studies were performed in a biphasic extractive and oxidative desulfurization (ECODS) system, composed by equivalent volumes of model diesel and extraction solvent (MeCN), using a H_2_O_2_/S ratio of 13 at 50 °C. The desulfurization process initiates with a liquid-liquid extraction for 10 min at 50 °C, in which non-oxidized sulfur compounds are transferred from the model diesel to the polar solvent MeCN. After this time, the reaching transfer equilibrium was achieved and the oxidant is then added to the ECODS system in order to proceed the sulfur removal from model diesel by an oxidative catalytic step. The results obtained for the ECODS systems catalysed by the different UiO-66(Zr) catalysts are shown in Figure 4. The initial extraction that occurred before the addition of the oxidant was similar between the different catalytic systems, achieving near 50% of extractive desulfurization just in 10 min. After this stage, the incessant desulfurization only occurs while performing the oxidative catalytic step, i.e., the sulfur compounds already present in the MeCN phase need to be oxidized to sulfide and/or sulfone, and consequently more sulfur compounds are relocated from the model diesel to the MeCN extraction phase.

The two catalysts prepared by the solvothermal method revealed different behaviour, with the UiO-66(Zr)-S1 revealing a considerable superior catalytic efficiency than the UiO-66(Zr)-S2. Using the first catalyst, near complete desulfurization was attained after just 3 h (99.5% of efficiency; only 10 ppm of sulfur from 1-BT remains in the model diesel). The utilization of the UiO-66(Zr)-S2 catalyst was not allowed ultra-deep desulfurization (S < 10 ppm) even at 4 h of reaction (80% of efficiency). These results suggest that the cool time of the prepared material in the oven may have an important influence in its catalytic performance, probably caused by the small structural differences that can occur and these play an important role in its catalytic efficiency for ECODS processes.

The desulfurization profiles obtained with the three UiO-66(Zr) catalysts prepared by MWAS are similar, and only a slightly higher catalytic efficiency has been observed for the UiO-66(Zr)-MW1 material during the first 1.5 h of reaction (94% of desulfurization efficiency). After 2 h of reaction, all the UiO-66(Zr)-MW samples revealed near 96% of desulfurization efficiency. After this time the desulfurization efficiency of MWAS catalysts did not increase appreciably (108 ppm of sulfur to 1-BT). These results indicate that the time of MWAS preparation of UiO-66(Zr) (60 or 15 min) and also the use of the modulator agent as AcOH, do not have a relevant influence in the catalytic efficiency of the obtained material. As previously reported in the literature, a faster preparation of UiO-66(Zr) may result in more defects in the MOF framework that may promote a higher catalytic efficiency [32].

From all the catalytic ECODS systems, the 1-BT was the most difficult sulfur compound to remove from model diesel and consequently, the most difficult to oxidize. The oxidative reactivity order observed, i.e., DBT > 4-MDBT > 4,6-DMDBT > 1-BT is well described in the literature and is related to the electronic density at the sulfur atom and some steric hindrance. [33,34,35] The possible mechanism involved in the oxidation of these sulfur compounds using the H_2_O_2_ oxidant, catalyzed by UiO-66(Zr), has been previously reported in the literature. [10,36] The active centers in UiO-66(Zr) framework are formed by the interaction of the oxidant with the Zr(IV) metal centers, originating Zr(IV)-peroxo groups on the surface of the material. In the next step, sulfur compounds are oxidized by the transfer of the oxygen atom from the Zr(IV)-peroxo group. More recently, Zheng et al. proposed the formation of active ^•^OH and ^•^O_2_^−^ radicals by the cleavage of O–O peroxo groups using temperature reaction [36].

**Figure 5:**
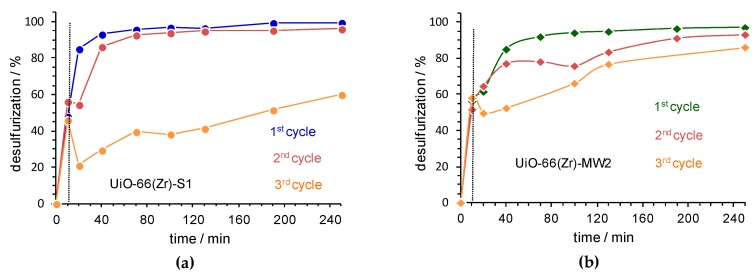
The desulfurization profile obtained the three ECODS cycles, catalyzed by UiO-66(Zr)-S1 (**a**) and UiO-66(Zr)-MW2 (**b**), using the biphasic system model diesel/MeCN (1:1) and H_2_O_2_/S=13, at 50 °C. The vertical dashed lines indicate the beginning of the catalytic step in the ECODS process by adding oxidant (H_2_O_2_).

The recycle capacity of solid UiO-66(Zr) catalysts was investigated for the most active catalyst, i.e., the UiO-66(Zr)-S1 and the material prepared by MWAS by the most sustainable and cost-effective procedure, i.e., UiO-66(Zr)-MW2. After each ECODS cycle, the desulfurized model diesel was removed and the solid catalyst was separated from the MeCN phase by centrifugation, washed with ethanol and dried at room temperature. The recovered catalyst was then used in a new ECODS cycle, using the same experimental conditions. The desulfurization results obtained for three consecutive cycles using the solvothermal UiO-66(Zr)-S1 (Figure 5a) indicate that the catalytic activity of this catalyst decreased drastically after the 2nd consecutive cycle. This catalyst presented a desulfurization efficiency of 99.5% for the 1st cycle, 95% for the 2nd cycle and 54% for the 3rd cycle, after 3 h of reaction. Interestingly, the recyclability studies using the UiO-66(Zr)-MW2 only revealed smaller differences of desulfurization profiles between ECODS consecutive cycles, mainly for reaction times inferior to 2 h (Figure 5b). After 3 h of reaction, the desulfurization efficiency is practically maintained for the three consecutive cycles (96% for the 1st cycle, 91% for the 2nd cycle and 85% after the 3rd cycle). In fact, using this MWAS catalyst, the deactivation observed for the 3rd ECODS cycle was minor, which may indicate a higher structural stability of the UiO-66(Zr) prepared by the MWAS processes, and can be promoted by the smaller particle size and the potential higher surface area of the MWAS samples.

**Figure 6:**
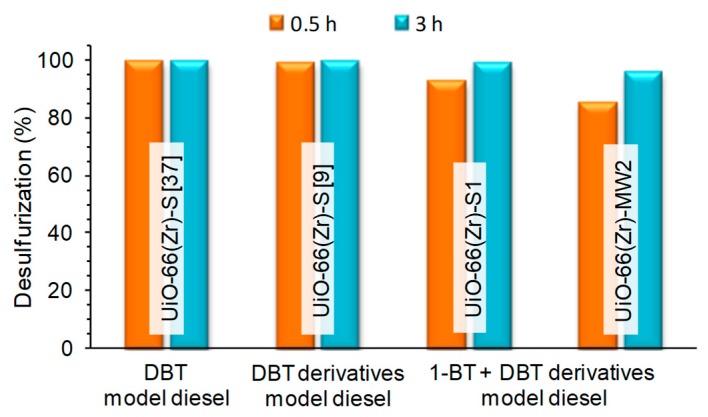
Desulfurization efficiency of UiO-66(Zr) catalysts used in different model diesel sulfur contents under similar experimental conditions (biphasic system model diesel/MeCN system, and H_2_O_2_ as oxidant at 50–60 °C [9,37].

Figure 6 compares the desulfurization efficiency to treat distinct model diesels containing different sulfur compounds: A single DBT model diesel [37] and two multicomponent model diesel, one formed by only DBT derivatives [9] and the other containing 1-BT and DBT derivatives (results of this work). All the treatments were performed using UiO-66(Zr) catalysts under similar ECODS experimental conditions (biphasic model diesel/MeCN system, H_2_O_2_ as oxidant and at 50 or 60 °C [37]). Approximately complete desulfurization was found after 0.5 h using model diesels without 1-BT. When this compound is present, near complete desulfurization is only achieved after 3 h. This important correlation is in accordance to the higher difficulty of oxidizing 1-BT than DBT derivatives. Furthermore, the loss of catalytic activity verified after the 2nd consecutive ECODS cycle with UiO-66(Zr)-S1 is promoted by the higher difficulty to desulfurize 1-BT and consequently to oxidize this compound.

The catalytic desulfurization efficiency of the UiO-66(Zr) catalyst prepared in this work by the solvothermal procedure (UiO-66(Zr)-S1) and by the fast preparation procedure using MWAS (UiO-66(Zr)-MW2) to treat the multicomponent model diesel with 1-BT and DBT derivatives is also compared in Figure 6. Only a slight difference of efficiency is observed after 0.5 h, having the MWAS catalyst 85% of efficiency instead of 93% obtained with the solvothermal material. This difference of activity observed is negligible when correlated to the advantage that the MWAS preparation procedure presents (only 15 min of reaction to prepare the catalyst). This result of the MWAS catalyst could not be directly compared with literature results since this is the first work reporting the application of MWAS UiO-66(Zr) in oxidative desulfurization systems.

### 3.3. Catalysts Stability

**Figure 7:**
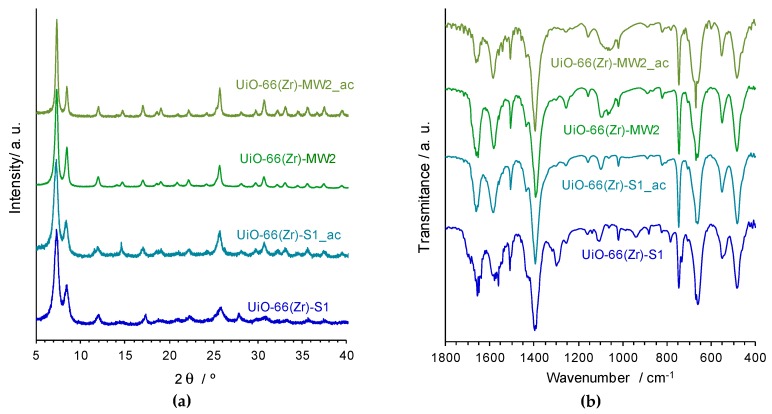
PXRD patterns (**a**) and FTIR-ATR spectra (**b**) of the UiO-66(Zr)-S1 and UiO-66(Zr)-MW2 materials in comparison with those after catalytic utilization [UiO-66(Zr)-S1_ac and UiO-66(Zr)-MW2_ac].

**Figure 8:**
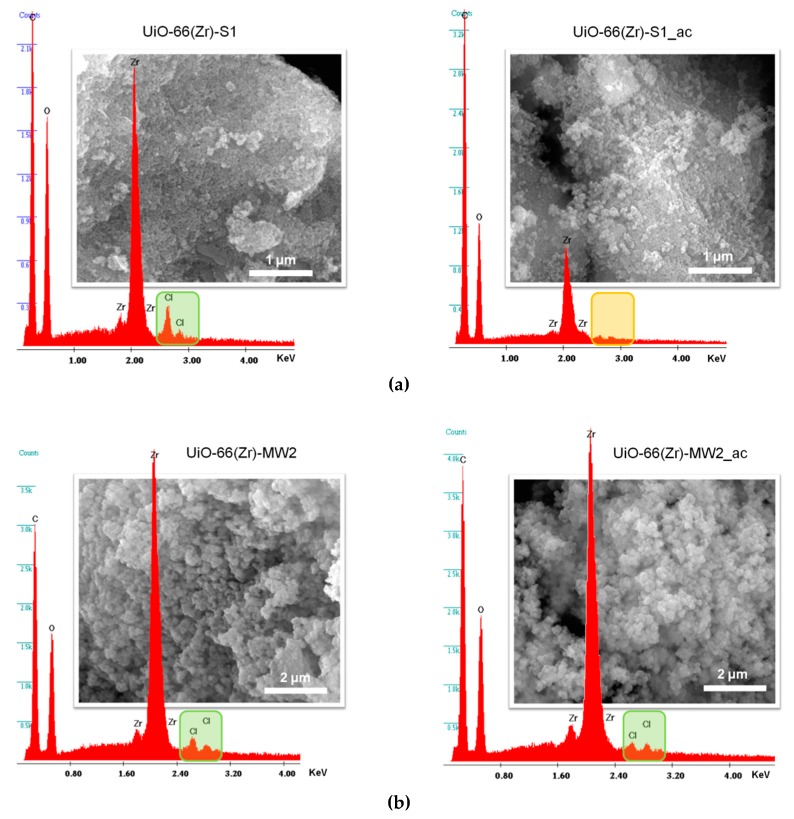
EDS spectra and SEM images of the UiO-66(Zr)-S1 (**a**) and UiO-66(Zr)-MW2 (**b**) materials in comparison with those after catalytic use [UiO-66(Zr)-S1_ac and UiO-66(Zr)-MW2_ac].

The structural robustness and chemical stability of the UiO-66(Zr)-S1 and UiO-66(Zr)-MW2 catalysts was evaluated by complementary characterization methods. The materials were recovered after the third ECODS catalytic cycle, UiO-66(Zr)-S1_ac and UiO-66(Zr)-MW2_ac (ac stands for after catalysis), and characterized by FTIR-ATR, PXRD and SEM / EDS (Figure 7). The PXRD patterns of both the materials after catalytic use exhibit indistinguishable profiles relative to the corresponding ones before catalysis (Figure 7a). All the main peaks remain practically unchanged regarding its positions and relative intensities, pointing to the maintenance of the MOF framework after the three ECODS catalytic cycles. This evidence is further supported by the FTIR-ATR data and SEM images, since the vibrational spectra of UiO-66(Zr)-S1_ac and UiO-66(Zr)-MW2_ac are similar to the respective before catalysis (Figure 7b), and significant morphologic changes are not observed in the MOF materials after their utilization in the catalytic reactions (Figure 8).

The apparent decrease in the catalytic performance of both materials UiO-66(Zr)-S1 and UiO-66(Zr)-MW2, particularly observed in the third ECODS cycle, cannot be attributed to the collapse of the crystalline structure or drastic structural changes in the framework of the MOF materials. Instead, this reduction of activity can be associated with the loss of the Cl from the MOF structure, since after catalytic use this element could not be identified by EDS spectrum of UiO-66(Zr)-S1_ac (Figure 8a). On the other hand, the Cl element is still presented in the EDS spectrum of the catalytic used UiO-66(Zr)-MW2_ac (Figure 8b). This also can explain the higher recycle capacity of the MWAS catalyst (Cl/Zr = 0.078 and 0.047 before and after three ECODS consecutive processes) compared to the solvothermal prepared material. As mentioned before, the potential existence of structural defect sites in the MOF framework results in charge and coordination deficiencies (open zirconium sites), which can be apparently compensated by Cl anions [10,31].

## 4. Concluding Remarks

The influence of the preparation method of the porous metal-organic framework (MOF) UiO-66(Zr) in their catalytic performance for the extractive and oxidative desulfurization system (ECODS) was evaluated. Two distinct samples of the porous MOF material UiO-66(Zr) were prepared by solvothermal synthesis [UiO-66(Zr)-S1 and UiO-66(Zr)-S2] and three different samples were isolated by microwave advanced synthesis (MWAS) [UiO-66(Zr)-MW1, UiO-66(Zr)-MW2 and UiO-66(Zr)-MWmod], following different preparation procedures. All these samples were structurally characterized and applied in the ECODS of a multicomponent model diesel containing 1-benzothiophene, dibenzothiophene, 4-methyldibenzothiophene and 4, 6-dimethyldibenzothiophene. A significant difference of catalytic activity was found between UiO-66(Zr) samples prepared by the solvothermal method, indicating that a fast cooling after the oven use is preferred than a slower cooling. The difference of Cl/Zr ratio found for these MOF structures indicates that a faster cooling probably promotes a higher incorporation of Cl anions in UiO-66(Zr) framework, what seems to suggest the presence of a higher number of defects in the structure and a higher catalytic activity. On the other hand, the UiO-66(Zr) materials prepared by MWAS using various reaction times (15 and 60 min) produced catalysts with similar Cl/Zr ratios and also particle sizes, which resulted in materials with identical catalytic efficiency. Furthermore, the addition of AcOH as a modular agent in the preparation procedure did not improve the material catalytic activity. Slightly higher oxidative desulfurization efficiency was found using the UiO-66(Zr)-S1 than the UiO-66(Zr)-MW2 material (99.5% of desulfurization was achieved after 3 h using UiO-66(Zr)-S1 catalyst, instead of 96% found for UiO-66(Zr)-MW2). However, the material prepared using the MWAS method showed higher recycle capacity in consecutive cycles. The loss of activity observed after the second cycle associated to the solvothermal material was not due to apparent structural modification or decomposition. However, this must be related to the loss of Cl content in the UiO-66(Zr)-S1 structure after catalytic use. Therefore, the preparation of UiO-66(Zr) material by the MWAS method conciliates various advantages associated with the shorter reaction time preparation and also higher catalytic stability.
